# Assessment of Biostimulation Methods Based on Chemical Communication in Female Doe Reproduction

**DOI:** 10.3390/ani12030308

**Published:** 2022-01-27

**Authors:** Paula R. Villamayor, Julián Gullón, Uxía Yáñez, María Sánchez, Pablo Sánchez-Quinteiro, Paulino Martínez, Luis Quintela

**Affiliations:** 1 Department of Genetics, Veterinary Faculty, Universidade de Santiago de Compostela (USC), Avda Carballo Calero s/n, 27002 Lugo, Spain; paulino.martinez@usc.es; 2 Department of Anatomy, Animal Production and Veterinary Clinic Science, Veterinary Faculty (USC), Avda Carballo Calero s/n, 27002 Lugo, Spain; pablo.sanchez@usc.es; 3 COGAL SL, Cuniculture Company, 36530 Rodeiro, Spain; julian.gullon@rai.usc.es (J.G.); cogal@cogal.net (M.S.); 4 Unit of Reproduction, Department of Animal Pathology, Veterinary Faculty (USC), Avda Carballo Calero s/n, 27002 Lugo, Spain; uxia.yanez@rai.usc.es (U.Y.); luisangel.quintela@usc.es (L.Q.)

**Keywords:** rabbit, biostimulation, reproduction, pheromones, urine, seminal plasma, chemocommunication, olfaction

## Abstract

**Simple Summary:**

Biostimulation is a natural technique employed in animal production to enhance reproductive parameters. In this study, we assessed the reproductive efficiency of female rabbits (receptivity, fertility, prolificacy and number of born alive and dead kits/litter) when exposed to different biostimulation conditions, which involved exposure to urine, seminal plasma or social separation between females, prior to artificial insemination (AI). Overall, despite all groups having showed a similar reproductive performance, our results indicated that female–female separation prior to AI could replace social interaction, and therefore reduce animal handling in farms (time–cost efficiency) with a consequent improvement of animal welfare. Future studies are needed to fully elucidate how chemical signals released through bodily secretions influence reproduction.

**Abstract:**

Biostimulation is an animal management practice that helps improve reproductive parameters by modulating animal sensory systems. Chemical signals, mostly known as pheromones, have a great potential in this regard. This study was conducted to determine the influence of short-term female rabbit exposure to different conditions, mainly pheromone-mediated, on reproductive parameters of inseminated does. Groups of 60 females/each were exposed to (1) female urine, (2) male urine, (3) seminal plasma and (4) female–female (F–F) separated, just before artificial insemination, and compared to a ‘golden method’ female–female interaction. The following reproductive parameters were analyzed for each group: receptivity (vulvar color), fertility (kindling rate), prolificacy and number of born alive and dead kits/litter. Our results showed that the biostimulation methods employed in this experiment did not significantly improve any of the analyzed parameters. However, female doe exposure to urine, especially to male urine, showed no significant higher fertility values (95.4%) when compared to the rest of the experimental conditions (on average 92.4%). Female–female interaction before artificial insemination, which is a common practice in rabbit farms, showed similar results as not establishing social interaction (F–F separated), which suggests that F–F interaction could be replaced by F–F separated, therefore avoiding unnecessary animal management and time cost. On the other hand, fertility ranges were lower for animals with a pale vulvar color whereas no differences were noticed among the other three colors which measure receptivity (pink, red, purple), thus suggesting that these three colors could be grouped together. Future studies should aim at determining potential chemical cues/pheromones released through bodily secretions that influence reproduction in rabbits, therefore contributing to animal welfare and to a natural image of animal production.

## 1. Introduction

Socio-sexual behaviors, such as fighting and mating, are essential for animal reproduction and survival [1,2]. In nature, individuals are continuously exposed to sensory signals from conspecifics and the environment, allowing them to communicate between themselves and modulate their behavior and reproductive physiology [3]. In high performance livestock, animals are usually kept indoors with less access to natural stimuli. Therefore, implementation of techniques based on interaction with natural cues has the potential, not only to increase their reproductive efficiency, but also to allow individuals to develop their own natural behavior, thus enhancing animal welfare.

Biostimulation is a natural technique employed in animal production to enhance reproductive parameters, and is based on modulating external environmental cues (visual, olfactory, pheromone, tactile, auditory, social and nutritional cues—among many others yet to be discovered) which elicit specific behavioral and endocrine responses in conspecifics [4,5]. Despite biostimulation methods usually entailing a mix of various external cues [6], pheromone signals play a pivotal role since they can trigger sexual behaviors by influencing reproductive physiology [7,8,9]. Indeed, the terms biostimulation and pheromone communication have been confusedly interchanged by the literature [6,10]. Pheromones are defined as chemical signals exchanged between organisms of the same species, causing a specific reaction in the receiver [11,12]. For instance, the sex pheromone ‘darcin’ is released in mice male urine and elicits sexual attraction of females [13]. These chemosensory cues are carried in bodily secretions (i.e., urine, seminal plasma) [14,15,16] and exocrine glands (i.e., lacrimal, mammary, mentonian, Harderian) [9,17,18].

Pheromone communication together with other visual and auditory cues participate in the biostimulation method called the ‘male effect’, in which females exposed to sexually active males trigger activation of luteinizing hormone (LH) secretion and synchronized ovulation [19]. In rabbits, the ‘male effect’ points to an improvement of doe reproductive performance [20] especially in does at first lactation [21]. However, little is known about the bodily secretions and pheromone cues involved in such behavior. Similarly, female–female interaction elicits a reproductive response in females after interacting between them [22]. In rabbits, despite studies to date not being conclusive at improving doe reproductive parameters by female–female interaction [23], placing together two females before artificial insemination (AI) has become an established routine as a biostimulation method in rabbit farms. Further studies are needed to validate the actual improvement of rabbit performance when two females are placed together before AI, especially due to the additional animal handling and greater workforce needed for this practice. Other biostimulation techniques such as lighting control [24], feeding control [25] and mother–litter separation [26] are also commonly used in rabbit farms.

Due to the induced-ovulation nature of female rabbits, various biostimulation methods are generally used in conjunction with hormone treatments to ensure ovulation and reproductive efficiency [27]. Gonadotropin-releasing hormone (GnRH) (or its analogues) is generally used at the time of insemination, either by intramuscular or intravaginal (into the insemination straw) administration [28,29,30] to induce luteinizing hormone (LH) peak triggering ovulation [31]. Recently, nano-drug delivery systems have emerged as a promising method to reduce GnRH dose in rabbit does [32]. Additionally, equine chorionic gonadotropin (eCG), intramuscular injection 48–72 h before AI, is also used to synchronize estrus [33] and it has been proven to increase receptivity, and prolificacy. However, repeated use of this hormone can induce immune response [34] and affect ovary function [35], with the consequent loss of reproductive efficiency [36]. Interestingly, several studies have shown that biostimulation methods (lighting and feeding programs, and/or mother–litter separation) could replace eCG administration [25,37,38], thus demonstrating that biostimulation methods are powerful tools that could potentially replace the use of hormones in rabbit farms.

Accordingly, the current study aimed to gain insights into the role of pheromone communication in rabbit doe reproduction. Specifically, our objective was to shed light on (1) the effect of female urine exposure, (2) male urine exposure, (3) seminal plasma exposure and (4) female–female (F–F) separation, when compared to F–F interaction, prior to AI, on improving the reproductive and productive performances in rabbit does.

## 2. Materials and Methods

### 2.1. Animals

This study was conducted according to the regulations and general recommendations of the National Board of Agriculture on the use of animals for scientific purposes. All the procedures were carried out under farm conditions in the industrial rabbit farm COGAL S.L. (Rodeiro, Spain). A forced ventilation system was used and the inside temperature was maintained between 18 °C and 22 °C using an air conditioned-heater system. All females were 3.5–4 kg weight from commercial hybrid (Hyplus strain PS19, Grimaud Frères, Roussay, France), and breeder males were 5–7 kg weight from Hyplus strain PS40. Males and females were located in separated farms.

### 2.2. Sample Collection

Urine: Pools of 330 mL of urine were obtained by ultrasound-guided cystocentesis from mature males and females (>180 days), 24 h before the behavioral experiment was performed, and kept at 4 °C overnight. Pure urine was used in all cases. Of note, by employing cystocentesis (the only method technically possible to ensure farm conditions), cues delivered in the lower urinary tract might be missed.

Seminal plasma: Obtained from an AI Center, 24 h prior to the behavioral experiment, from 60 mature males (>180 days). All ejaculates were mixed together and centrifuged at 3000 rpm, 10 min, to obtain the seminal plasma, which were then kept at 4 °C overnight. Before use, it was diluted 1:3 in Ringer Lactate Solution.

### 2.3. Semen Processing and Artificial Insemination

To perform the AI, semen was collected with artificial vagina and stored at 16 °C before use within a 24 h period. Once the ejaculates were collected, they were pooled and diluted with a commercial extender (MRAbit^®^ (Alarelin); Kubus SA, Madrid, Spain) to a standard concentration of 60 × 10^6^ spermatozoa/mL. Does were vaginally inseminated using disposable plastic pipettes, receiving a dose of 30 × 10^6^ spermatozoa in a volume of 0.5 mL.

### 2.4. Reproductive Managemenent

All does employed for the behavior experiment were between third and ninth kindling and were evenly distributed among the five experimental groups (see ‘Experimental Design’ in M & M). Nulliparous (first kindling) and primiparous (second kindling) were not considered for the study due to their reproductive performance instability—they show the best and worse reproductive performance, respectively, among all kindling numbers. None of the animals were treated hormonally with eCG to synchronize estrus. All does were inseminated on day 11 after parturition and were lactating a maximum of 11 kits. Sexual receptivity was confirmed by determining the color of the vulva (pale, pink, red, purple) at the time of AI [25]. Pregnant or lactating does were fed ad libitum whereas non-pregnant or non-lactating does were restricted to 150 g/day of commercial food except in the period from 6 days before AI to the day of pregnancy diagnosis, during which they were also fed ad libitum. Light intensity was 70 lux, with an artificial lighting program of 12 h (light) L/12 h (dark) D, which was changed to 16 h L/8 h D 6 days before does AI. After AI, light hours were decreased 1 h/day during 4 days until coming back to the normal program. Controlled suckling was applied to all does from 0 to 10 days post-partum, by keeping the nest door closed and only opening it every 24 h for 5–10 min, to allow the kits to suck once a day. On the day of AI (day 11 post-partum), suckling was 6 h delayed, until 5–10 min before performing the AI. This made a 30 h mother–litter separation. From day 12 post-partum (i.e., 1 day after AI) to weaning (30–35 days post-partum), free suckling was allowed by keeping the nest door open. At 11–14 days after AI, all does were diagnosed for pregnancy by transabdominal palpation. Parturitions took place mainly on day 30 post-AI. When all does completed parturition, the prolificacy and number of born alive and dead kits/litter were recorded. Then, the number of rabbits per litter was adjusted to 11 kits of equal body size.

### 2.5. Experimental Design

We conducted a behavioral experiment in does to determine whether female reproductive parameters (receptivity, fertility, prolificacy and number of born alive and dead kits/litter) vary according to four given conditions: (1) Exposure to female urine, (2) Exposure to male urine, (3) Exposure to seminal plasma and (4) Female–female (F–F) separated, compared to a ‘golden method’ (F–F interaction). We consider as ‘golden method’ the most common practice in the analyzed farm, which consists in placing together two females 10 min before AI. The experiment was repeated three times (three time points), every 42 days (in three consecutive inseminations). Each of the five groups were composed of ~60 does in the first trial, and in such a first time point, all animals were lactating does between third and seventh kindling. Note that the same animals were kept during the three time points, and therefore in the second and third time points, some does could be non-lactating—if they were not pregnant, also called ‘negative does’—; these animals were not considered for the statistical analysis. Additionally, some animals were eliminated during the experiment, mainly due to health reasons during the peripartum, and they were also removed for the statistical analysis. When possible, they were replaced by other animals under the same conditions. A total of 734 litters were evaluated, all of them individually monitored for each of the reproductive parameters analyzed.

For conditions 1, 2 and 3, the corresponding stimulant was sprayed around the nose area, 1 h, 15 min, and 1 min before insemination. There was no direct contact between the worker and the animal to avoid stress and discard changes in reproductive parameters due to animal manipulation. Specifically, 1 mL nasal spray was used in each exposure per animal, in total, 3 mL/individual. Additionally, urine drenched wool was hung in the cages after the first exposure to ensure the permanent exposition of the animal to the stimulant—some animals gnawed it. AI and the corresponding handling were always performed by the same farm workers to reduce statistical noise. Of note, chemical cues could be detected by the olfactory epithelia but also by the vomeronasal organ, since this latter opens in the anterior part of the nasal cavity [39].

### 2.6. Statistical Analysis

Statistical analysis was performed using SPSS 20.0 software (SPSS Inc., Chicago, IL, USA). Data on receptivity and kindling rates were analyzed by χ^2^ and prolificacy and number of born and dead were analyzed using analyses of variance (Anova procedure), considering the distributions of the variables.

A binary logistic regression was performed with fertility (yes/no) as dependent variable, while univariate general linear models (GLM) were performed with total born, alive and dead kits/litter as dependent variables, in both analyses, taking the number of insemination (1st, 2nd and 3rd), the number of kindling (1st, 2nd, etc.), the experimental group (urine female, urine male, etc.), and receptivity (vulva color) as independent variables. These analyses aimed to determine the factors that influence fertility and prolificacy, respectively. For the logistic regression, the most predictable variables were tested by using the method “Backward conditional” Hosmer and Lemeshow test (*p*-value > 0.5).

In all cases, differences were considered statistically significant at *p* < 0.05 level.

## 3. Results

We estimated the reproductive parameters of a total of 734 female does (receptivity, fertility, prolificacy and number of born alive and dead kits/litter) (Table 1) when they were exposed to different biostimulation conditions: exposure to either female or male urine and seminal plasma as potential source of pheromones, and also physical separation (F–F separation) compared to F–F interaction (golden method in the farm), during the 10 min before AI.

### 3.1. Fertility

**Figure 1:**
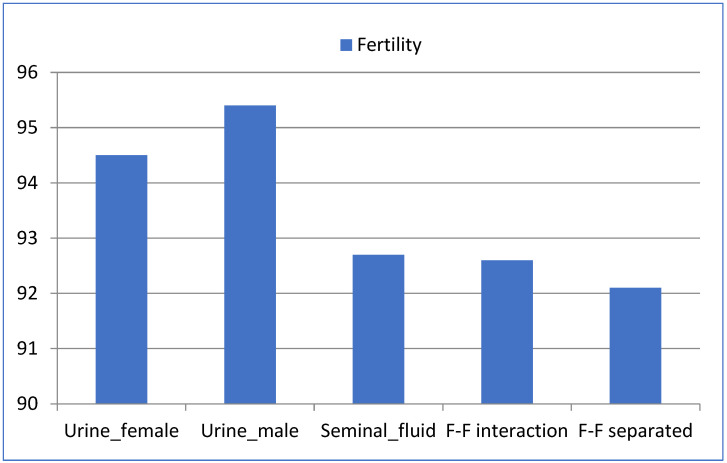
Fertility (kindling rate) in each of the different experimental groups.

When assessing fertility (kindling rate) (Table 2), we found no significant differences between experimental groups (Figure 1). However, females exposed to urine, especially those exposed to male urine, showed slightly higher fertility levels, although not significant (*p* value > 0.05). 

In the third insemination, female does showed significantly higher fertility levels (*p* value < 0.01) than in the first and second inseminations (Table 2). It should be noted that in all cases, fertility rate was above 90%, and a range between 85–98% lies within the usual rate of this farm depending on different reasons (i.e., animal management, diet, environmental and external factors, etc.).

**Figure 2:**
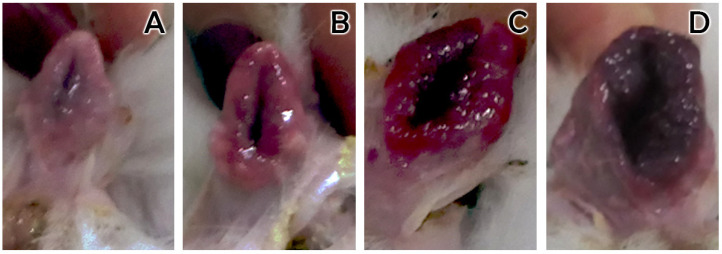
Vulvar color of rabbit doe. (**A**): pale; (**B**): pink; (**C**): red; (**D**): purple.

On the other hand, receptivity rate measured by vulvar color (Figure 2) did not show a significant impact on fertility (*p* value > 0.05), even though our results point to higher fertility rate in females with purple vulvar color (97.2%) than those with pale vulvar color (90%) (Table 2). Finally, fertility rate seems to be not influenced by kindling number, despite females in their 8th and 9th kindling showed higher fertility rate. This could be an artifact, especially because a significant fewer number of animals were considered for these two kindling. Of note, in the first trial there were only animals from 3rd to 7th kindling, and only in the second and third trials animals of 8th and 9th kindling, respectively were employed.

### 3.2. Prolificacy

**Figure 3:**
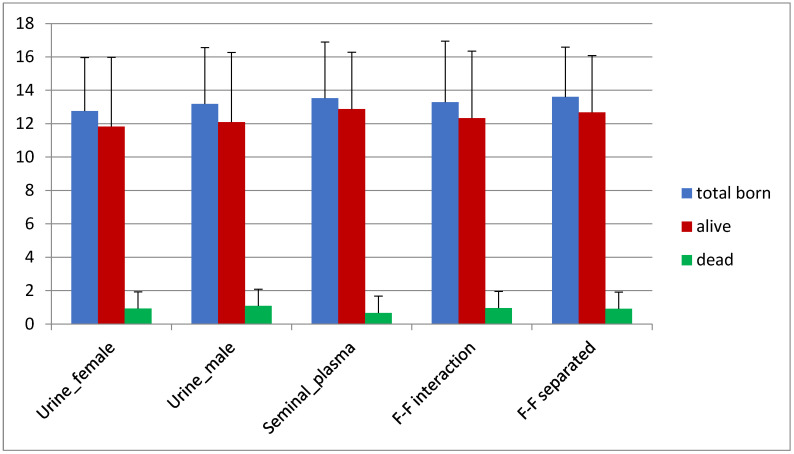
Prolificacy rate (number of born alive/dead animals) in each of the different experimental groups.

We found no prolificacy differences between the five experimental groups (Figure 3). The mean of the total born animals considering all the conditions presented in Table 2 (experimental group, insemination number, vulvar color, kindling number) was 13.04 ± 3.36 (12.16 ± 3.86 alive and 0.88 ± 2.25 dead). This indicates that female exposure to urine of both females and males does not have an impact in prolificacy. Similarly, F–F interaction and F–F separated showed similar results. Prolificacy was significantly different between second and third insemination (*p* value < 0.05), which highlights the importance of considering different insemination times due to physiological reasons or farm conditions.

**Figure 4:**
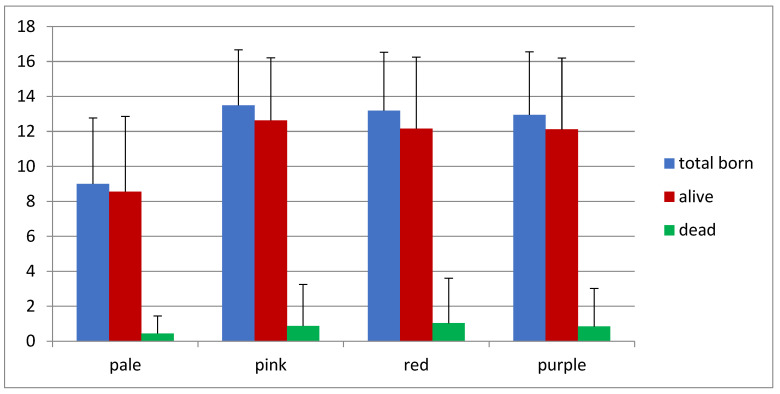
Prolificacy rate depending on the receptivity (vulvar color).

Moreover, we did find significant differences in the prolificacy rate depending on the receptivity (Figure 4). Four vulvar colors have been previously described to assess receptivity rate: pale, pink, red and purple (Figure 1), showing increasing levels of receptivity [25]. We saw that females showing pale vulvar color at the time of AI had a significant reduced number of total born kits when compared to females with pink and red vulvar colors (*p* value < 0.01). Our data confirmed that prolificacy depends on sexual receptivity, but only when considering ‘pale’ with ‘lower prolificacy’ vs. ‘not-pale or the sum of pink, red and purple’, with ‘higher prolificacy.

### 3.3. Receptivity

**Figure 5:**
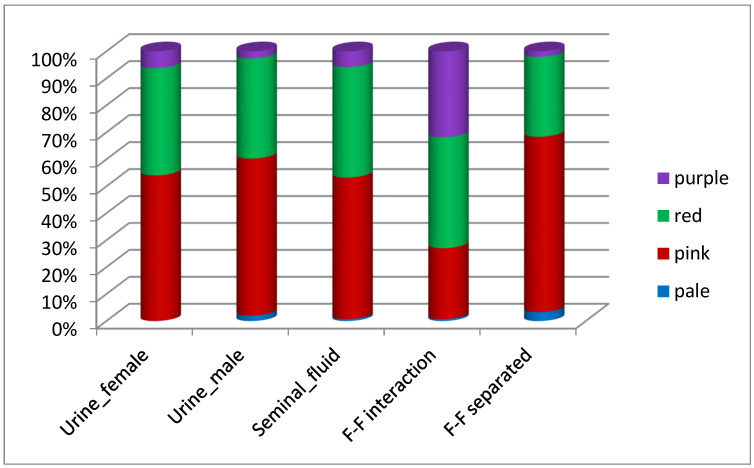
Receptivity rate (vulvar color) depending on the experimental group. Among variables statistical comparisons.

When looking at the receptivity rate (vulvar color), the experimental group ‘F–F interaction’ showed the highest purple vulvar color (Figure 5), but importantly, this did not affect fertility and prolificacy parameters, as previously explained. As a qualitative estimation, we also found a strong ‘riding behavior’ in this group when the two does were placed together before AI. On the other hand, the group that showed the highest percentage of pale vulvar color was ‘F–F separated’ (3.3% of all ‘F–F separated’) (Figure 4). This percentage is quite low and overall, only 10 out of the 734 (1.3%) individuals used for the analysis showed pale vulvar color, which indicates successful levels of female estrus synchronization in the farm.

We also performed a binary logistic regression to estimate the relationship between fertility levels and the experimental group, number of insemination, vulvar color and number of kindling. The results were similar to those obtained with the chi-squared test. The experimental group did not influence fertility, and the only significant independent variable was the insemination number (Table 3). Note that vulvar color did not significantly influence fertility in this model likely due to the low number of females presented with pale vulvar color—no statistical power.

Finally, significant results (*p* value < 0.05) were obtained in the univariate general lineal model (GLM) analysis when only considering ‘prolificacy/number of born alive and dead kits/litter’ as dependent variables. Considering as dependent variable ‘born dead kits’, we found significant influence with the interaction between ‘experimental group’, ‘vulvar color’ and ‘number of kindling’. Considering as dependent variable ‘born alive kits’, we found significant influence only with ‘vulvar color’. Considering as dependent variable ‘total born’, we found significant influence with ‘vulvar color’ and also with the interaction between ‘insemination number’, ‘experimental group’ and ‘vulvar color’. Accordingly, the GLM analysis confirmed a significant influence between prolificacy levels and receptivity rate (vulvar color), insemination number and experimental group.

## 4. Discussion

We found high percentages of fertility and prolificacy in female reproduction by using different biostimulation methods, despite no hormones (eCG) were employed. Using the F–F interaction group as a ‘golden method’, we determined that F–F separated individuals prior to AI show similar fertility and prolificacy levels to the F–F interaction group. Therefore, we determined that F–F separated is beneficial compared to F–F interaction, since it is less invasive, less expensive and more natural—avoidance of animal manipulation (no animal stress) with a consequent reduce in workforce (time and cost). Additionally, we did not find any significant improvement in female rabbit performance when they were exposed to female urine, male urine or seminal plasma.

### 4.1. Social Interaction Seems Not Influencing Reproductive Physiology in Farm Female Doe

In nature, individuals interact among them influencing their reproductive physiology [40]. The biostimulation method ‘male effect’ has been a valuable management tool exploited in small ruminants [19,41,42] and swine [43] husbandry to stimulate the onset of puberty and to reduce the postpartum period. In cattle, ‘male effect’ has received little attention, and even though bull–cow interaction has proved to influence female reproductive activity [44], the literature is not consistent [6,43], and therefore, this practice has not yet been implemented as a common farm routine. In rabbits, ‘male effect’ appears to slightly improve doe reproductive performance [20]. However, significant effects were only found in does at first lactation [21] and published data have been contradictory [45,46], hampering consistent conclusions. 

Females also elicit a reproductive response to female–female interaction, and female chemical signals play important roles in sexual attraction [47]. Specifically, reproductive response to F–F interaction has been shown in goat [48], wild boar [49], human [50] and beef cow [51]. In rabbits, we found an increase in receptivity rate (vulvar color) in the experimental group F–F interaction (highest number of females with purple vulvar color (Figure 4)) but importantly, this did not affect fertility and prolificacy parameters. Additionally, we also found strong ‘riding behavior’ in the F–F interaction group (qualitative estimation) which could confuse operators, who might associate such behavior to higher fertility and prolificacy rates. Considering that F–F interaction is used as a common biostimulation method in rabbit farms, we argue that since no differences in fertility and prolificacy rates were noticed between experimental groups in our study, such management should be reconsidered in order to reduce animal handling and a substantial time cost. Likely this management might offer better results in farms with lower fertility, where there is higher room for improvement.

### 4.2. Urine as a Potential Source of Sex Pheromones in Female Doe Reproduction

Despite no significant differences were found between experimental groups, females under male and female urine exposure before AI reached the highest levels of fertility, especially when exposed to male urine (95.4% with male urine and 94.5% with female urine, compared to 92% in the rest of the groups). 

Urinary pheromones have been largely studied in mice and are known to influence sexual behavior [13,52]. Indeed, the ‘Whitten effect (1958)’ [53] refers to female estrus synchronization when they are exposed to male urine [54]. In farm animals, urine has been shown to accelerate puberty in cattle [55], whereas in goats it did not improve reproductive parameters [56]. Interestingly, females have preference towards urinary pheromones of dominant mice, but not towards subordinate ones [57]. In our experiment, we did not consider differences between dominant and subordinate males since urine from both was pooled. Further studies should consider only urine from dominant males.

### 4.3. Seminal Plasma Might Arise as a New Source of Pheromones

Although not as widely known as urine, seminal plasma might also be a reliable source of pheromones. In rabbits, a lipocalin was found in seminal plasma, showing significant similarity with ‘urinary’ and ‘salivary’ pheromone carriers [14]. More recently, [15] Scott et al. (2019) identified a sex pheromone in seminal plasma of sea lamprey. We did not find significant improvement of female doe reproduction when exposed to seminal plasma before AI. However, we should consider that seminal plasma was diluted 1:3 and a higher concentration might render better results. Similarly, it might be the case where only dominant males release these molecules, and therefore pheromone power has got diluted by pooling seminal plasma from all males.

Interestingly, seminal plasma is known to contain an ovulation-inducing factor (OIF) in several species [58,59] including rabbits [60]. OIF has been identified as a β neurotrophin (β-NGF) [58], which modulates ovulation [61], and has been suggested to have direct action on GnRH neurons outside the blood barrier [59]. Intramuscular injection of seminal plasma containing β-NGF showed a positive effect in llama but not in rabbit ovulation [60]. However, adding β-NGF to seminal dose has been proposed to replace GnRH in rabbit reproduction [62,63]. Despite their site and mechanism of action are unknown, β-NGF action appears to involve hypothalamic kisspeptin neurons [61]. Since male odors detected through the vomeronasal organ (main pheromone-receiver organ) are known to activate kisspeptin neurons in female mice [64], we argue that β-NGF might act as a pheromone or pheromone carrier, which triggers activation of kisspeptin neurons in the central nervous system and modulate ovulation. Further studies should be performed to determine whether female nasal exposure to OIF activates vomeronasal and hypothalamic kisspeptin neurons, and ultimately influences ovulation. Importantly, understanding the female response to seminal plasma will eventually shed new light on human infertility and pregnancy disorders [65].

### 4.4. Practical Considerations When Assessing Biostimulation Methods

This study was performed in female rabbits between 3rd and 9th kindling. Nulliparous and primiparous females were excluded due to their unstable reproductive parameters. Previous literature clearly indicates that nulliparous females have the best fertility rates, while primiparous females show the lowest values for this parameter [66]. The reason for this seems to be related to the energy balance during postpartum: nulliparous females do not present a negative energy balance as they are not lactating, while primiparous have lactation and also growth needs, with a considerable negative energy balance. Interestingly, previous studies have shown that primiparous does are the only group to significant respond to ‘male effect’ [21]. This might indicate that biostimulation methods become efficient in animals with lower conception rates. Further studies should consider only primiparous females, where either ‘male effect’ or F–F interaction might help improve their reproductive rates.

The effectiveness of biostimulation methods or hormonal treatments depends on the basic performance—physiological, health and behavioral states—of does at the time of AI [67]. Previous reports on rabbit reproduction management have shown that biostimulation or hormonal methods improve reproductive performance in females with 50–60% of average conception rate. However, as the percentage of conception rates increased, the methods employed became less efficient, and with average conception rates of 75–80%, the difference between the control and treated groups (either biostimulation or hormonal methods) was non-significant [68].

It should be taken into account that the farm employed for our experiment has considerably overall high fertility (usually higher than 90%) and prolificacy levels, becoming difficult to achieve significant improvement of any reproductive parameter. Our experimental framework emphasizes the importance of large sample size and biological replications to obtain reliable data that allow the detection of statistical differences between the treatments and the golden method. To test biostimulation efficiency, further studies should consider the same experimental approach in farms with lower fertility rates (50–60%).

### 4.5. Could Biostimulation Methods Reduce or Replace Hormonal Treatments?

Despite hormonal treatments not being tested in our study, it is clear that biostimulation methods may have potential as an alternative approach to exogenous gonadotropins to improve sexual receptivity and, consequently, the overall productivity of rabbit farms [21]. This is especially important in the context of a society where consumers are against the use of hormones in animal production [68], wishing to buy products labeled as produced with no added hormones [69].

Female rabbit ovulation does not occur spontaneously, and even though the ovulation triggered mechanism is unknown, coitus-related stimulus results in a rise in circulating luteinizing hormone (LH) that causes ovulation [31]. Therefore, in rabbit farms, this hormonal state has to be artificially induced. Currently, the most frequently used method is either intramuscular or intravaginal (into the insemination straw) administration of GnRH (or its analogues) at the time of AI [28,29,30], and recently, new nano-drug delivery systems have been proposed as a new method for reducing hormone dose and improving AI efficiency [32]. However, even though human exposure to GnRH after rabbit meat consumption is negligible (EMA/CVMP/156095/2017) [70], a report on minimum standards for the protection of farm rabbits (2016/2077(INI)) [71] highlights the importance of meeting high standards of animal health and welfare, where natural biostimulation techniques are encouraged to be implemented to reduce and potentially replace the use of exogenous hormones.

Similarly, eCG injection has proven to improve productivity [33] but biostimulation methods such as mother–litter separation could reduce its use [37]. Future experiments considering a hormone-treated group are needed to verify whether eCG could be replaced by any of the biostimulation methods shown here, both in farms with high reproductive performance, and also in farms with lower reproductive parameters. All in all, further research in the field should aim at replacing hormones with different biostimulation methods, thus enhancing animal welfare and greatly improving the image of our industries in society.

## 5. Conclusions

All the experimental groups showed high percentages of fertility and prolificacy, despite no hormones (eCG) were employed. F–F interaction prior to AI (‘golden method’) could be replaced by F–F separation, since it is less invasive and more time–cost efficient. Additionally, female doe exposure to male and female urine and to seminal plasma did not show any significant increase in the reproductive parameters analyzed when compared to the golden method. However, future experiments should not discard urine as a potential source of reproductive-related cues. Further studies should approach primiparous females (lower reproductive levels) and farms with lower fertility rates (50–60%), where there is more room for improvement. In addition, biological fluids used as a source of pheromones should be especially obtained from dominant males. Finally, biostimulation methods based on chemical communication might arise as potential sources to reduce or replace current hormonal treatments, and therefore contribute to animal welfare and to a natural image of animal production.

## Figures and Tables

**Table 1:** Reproductive parameters analyzed with their corresponding definition and way of measuring.

Parameter	Definition	Measurement
Receptivity	Behavioral receptivity to mating, which indicates estrus	Vulvar color (pale, pink, red, purple)
Fertility	Average number of females that give birth successfully	Percentage of females that give birth (kindling rate)
Prolificacy	Average number of born kits/litter	Number of born kits/litter
Number of born alive kits/litter	Average number of born alive kits/litter	Number of born alive kits/litter
Number of born dead kits/litter	Average number of born dead kits/litter	Number of born dead kits/litter

**Table 2:** Fertility (kindling rate), prolificacy (total born/litter) and number of born alive and dead kits/litter considering experimental group, insemination number, vulvar color and kindling number. N: number of positive/total female does. SD: standard deviation. Different letter in the same column indicates *p*-value < 0.05.

Experimental Group	N (Fertility)	Fertility%	N (Prolificacy)	Prolificacy ± SD	Alive ± SD	Dead ± SD
Urine_female	138/146	94.5	138	12.76 ± 3.2	11.83 ± 4.15	0.93 ± 2.38
Urine_male	144/151	95.4	144	13.18 ± 3.38	12.09 ± 4.18	1.09 ± 2.79
Seminal_plasma	127/137	92.7	127	13.53 ± 3.36	12.87 ± 3.41	0.67 ± 1.99
F–F interaction	137/148	92.6	137	13.29 ± 3.65	12.33 ± 4.02	0.96 ± 2.35
F–F separated	140/152	92.1	140	13.6 ± 2.99	12.68 ± 3.4	0.92 ± 2.49
Insemination Number						
1	234/259	90.3 ^a^	234	13.46 ± 3.73 ^abc^	12.35 ± 4.2	1.11 ± 2.39
2	224/244	91.8 ^a^	224	13.55 ± 2.97 ^ab^	12.72 ± 3.44	0.83 ± 2.15
3	228/231	98.7 ^b^	228	12.78 ± 3.19 ^ac^	11.98 ± 3.88	0.81 ± 2.7
Vulvar Color						
Pale	09/10	90	9	9 ± 3.77 ^a^	8.56 ± 4.3 ^ac^	0.44 ± 1.01
Pink	352/377	93.4	352	13.49 ± 3.18 ^b^	12.63 ± 3.58 ^b^	0.87 ± 2.38
Red	256/276	92.8	256	13.19 ± 3.34 ^b^	12.16 ± 4.09 ^b^	1.03 ± 2.58
Purple	69/71	97.2	69	12.95 ± 3.61 ^b^	12.12 ± 4.08 ^c^	0.84 ± 2.18
Kindling Number						
3	52/56	92.9	52	14.13 ± 4.17	13.15 ± 4.32	0.98 ± 1.84
4	144/157	91.7	144	13.34 ± 3.27	12.58 ± 3.88	0.76 ± 2.26
5	126/138	91.3	126	13.53 ± 3.13	12.94 ± 3.46	0.6 ± 1.8
6	120/130	92.3	120	13.4 ± 3.48	11.89 ± 4.42	1.51 ± 3.29
7	120/128	93.8	120	12.7 ± 3.4	11.77 ± 3.78	0.93 ± 2.27
8	85/86	98.8	85	13.03 ± 2.69	12 ± 3.47	1.04 ± 2.86
9	39/39	100	39	12.82 ± 3.33	12.44 ± 3.36	0.38 ± 1.13

**Table 3:** Final model of the binary logistic regression. Dependent variable: fertility (kindling rate). The only predictable independent variable was the number of insemination (*p*-value = 0.03).

Variable	Values	OR (Ods Ratio)	Confidential Interval for OR	*p*-Value
Insemination	first	reference		
	second	1.2	0.646–2.215	0.568
	third	8.12	2.418–27.266	0.001

## Data Availability

The data presented in this study are available within the article. All relevant data are within the manuscript, and are fully available without restriction.
